# Effects of Pile-Fermentation Duration on the Taste Quality of Single-Cultivar Large-Leaf Dark Tea: Insights from Metabolomics and Microbiomics

**DOI:** 10.3390/foods14040670

**Published:** 2025-02-16

**Authors:** Wanying Yang, Ruohong Chen, Lingli Sun, Qiuhua Li, Xingfei Lai, Zhenbiao Zhang, Zhaoxiang Lai, Mengjiao Hao, Qian Li, Sen Lin, He Ni, Shili Sun

**Affiliations:** 1 Guangdong Provincial Key Lab of Biotechnology for Plant Development, School of Life Sciences, South China Normal University, Guangzhou 510631, China; ywy000430@163.com; 2 Tea Research Institute, Guangdong Academy of Agricultural Sciences/Guangdong Provincial Key Laboratory of Tea Plant Resources Innovation & Utilization, Guangzhou 510640, China; chenruohong@gdaas.cn (R.C.); sunlingli@tea.gdaas.cn (L.S.); liqiuhua@tea.gdaas.cn (Q.L.); laixingfei@tea.gdaas.cn (X.L.); zhangzhenbiao@tea.gdaas.cn (Z.Z.); laizhaoxiang@tea.gdaas.cn (Z.L.); haomengjiao@gdaas.cn (M.H.); 3 Guangdong Academy of Agricultural Sciences, Sericultural & Agri-Food Research Institute/Key Laboratory of Functional Foods, Ministry of Agriculture and Rural Affairs/Guangdong Key Laboratory of Agricultural Products Processing, Guangzhou 510610, China; liq@gdaas.cn (Q.L.); linsen@gdaas.cn (S.L.)

**Keywords:** dark tea, quality, taste, pile-fermentation, metabolomics, microbiomics

## Abstract

The pile-fermentation conditions and raw materials used play a vital role in determining the stability and quality of dark tea. In this study, sensory quality evaluation, metabolomics, and microbiomics techniques were used to investigate the effect of pile-fermentation duration on the taste quality of single-cultivar large-leaf dark tea (SLDT) and its underlying metabolite and microbial mechanisms. The study revealed that a 60-day duration resulted in a better SLDT sensory quality, with astringency and bitterness significantly reduced and sweetness increased. Catechins and theaflavins with ester structures, L-epicatechin, methyl gallate, protocatechuic acid, gallic acid, salicin, chlorogenic acid, and neochlorogenic acid were key taste metabolites contributing to the reduction of astringency and bitterness. Salicylic acid and D-sorbitol helped form the sweetness. Correlation analysis found out *Aspergillus*, *Thermomyces*, *Bacillus*, *Staphylococcus*, and *Micrococcaceae* were core functional microorganisms linked to these metabolites, helping to foster the higher quality of SLDT. Microorganisms shaped the taste quality of SLDT through metabolic processes and enzyme secretion during pile-fermentation. This study provided insights into the metabolite basis and microbiological mechanisms of SLDT taste formation and offered guidance for optimizing production processes to improve the stability and quality of dark tea.

## 1. Introduction

Dark tea is increasingly popular among consumers, especially in China and Southeast Asia, for its pleasant sensory feeling and numerous health benefits, such as antioxidant, anti-obesity, and anti-diabetic functions [1]. Unlike other tea types such as green tea, oolong tea, and black tea, dark tea has unique sensory qualities, including a black-auburn appearance, red–brown infusion, a hint of stale and fungus fragrance, and a mellow, sweet, and smooth taste, occasionally accompanied by slight bitterness and astringency [2]. Notably, the dark tea industry is now facing a problem, i.e., commercially available dark teas are mainly made from traditional group tea plant varieties, which can lead to unstable quality of each batch of dark tea [3]. Therefore, it is necessary to use a single-cultivar material to produce dark tea.

The processing of dark tea generally consists of four key steps: green-removing, pile-fermentation (PF), steaming, and drying, with its hallmark PF process representing the linchpin for the formation of its unique quality [4]. Conditions such as temperature, humidity, and duration of the PF process can cause changes in the microbial communities, among which the duration has been considered to be the most important factor and usually spans 1 to 2 months [5,6]. Under microbial interactions, changes in PF duration can lead to the transformation of metabolite components, ultimately affecting the quality of dark tea [7].

Taste is the key factor for evaluating the organoleptic quality of tea, which mainly includes bitter, astringent, fresh, sour and sweet flavors [8]. The flavor characteristics of dark tea are dominated by bitterness, astringency, and sweetness, which are mainly dependent on the levels of flavor compounds, including flavonoids, phenolic acids, amino acids, alkaloids, and soluble sugars [9]. A suitable PF duration can alter the levels of these flavor compounds, thereby conferring a better flavor profile to dark tea [5]. On the contrary, inadequate PF duration may result in the development of sour and bitter flavors in dark tea [10]. However, the effect of PF duration on the taste and its metabolites basis of single-cultivar large-leaf dark tea is unclear.

Microbial action is considered to be the key to the PF process of dark tea [11]. Fungi such as *Aspergillus*, *Debaryomyces*, *Rasamsonia*, and *Thermomyces*, as well as bacteria like *Enterobacteriaceae*, *Bacillaceae*, *Lactobacillaceae*, and *Pseudonoccardiaceae*, are frequently identified as dominant microorganisms during the PF process of dark tea, which can promote the metabolite reactions [12]. For example, green tea leaves fermented by *Aspergillus niger* formed a red–brown tea infusion by increasing theabrownins and reduced astringency and astringent aftertaste by decreasing catechins [13]. However, previous studies on microbial transformation primarily used traditional dark tea made from group tea plant varieties, characterized by unstable metabolite profiles and varying microorganisms [14]. It is more conducive to elucidating the taste formation mechanism using dark tea derived from a singular raw material [6].

Metabolomics and microbiomics are core tools in the field of food quality research [15,16]. To investigate the quality profiles, taste compounds, microbial communities, and their metabolic potentials of single-cultivar large-leaf dark teas under different PF durations, sensory quality evaluation, metabolomics, and microbiomics techniques were used to study the tea samples with 0, 10, 20, 30, 40, 50 and 60 days of PF duration, respectively. The study revealed that a 60-day duration resulted in better taste quality than other durations, and microorganisms shaped the taste through metabolic processes and enzyme secretion during pile-fermentation. This study can provide a theoretical foundation of the metabolite basis and microbial mechanisms underlying taste formation of single-cultivar large-leaf dark tea and offer guidance for optimizing production processes to improve the stability and quality of dark tea.

## 2. Materials and Methods

### 2.1. Chemicals and Reagents

Methanol, acetonitrile, and ethanol of chromatographic purity were procured from Merck (Darmstadt, Germany). Chromatographically, pure standards were purchased from BioBioPha (Kunming, China) or Sigma-Aldrich (St. Louis, MO, USA). The Trace Colony DNA Extraction Kit, KAPA Library Amplification Kit, Nextera XT Index Kit, and KAPA Pure Beads Kit were sourced from Nanxin (Guangzhou, China). Yinghong No. 9 sun-dried summer green tea was supplied by HuaXia Dream Co., Ltd. (Qingyuan, China).

### 2.2. Tea Samples Collection

Yinghong No.9 large-leaf tea variety has high tea polyphenol content and is a suitable raw material for making dark tea [17]. Yinghong No.9 sun-dried green tea with 1 bud and 2–3 leaves was used as the raw material for the processing of single-cultivar large-leaf dark tea (from 8 July to 13 September 2019). The PF process followed the processing procedure outlined in the Guangdong Sinking Tea Production Standard DB44/T 1248-2013. Firstly, piling 500 kg of sun-dried raw green tea in a fermentation room, then evenly spraying it with 20% of its weight of water. The pile was turned over and covered with a moisturizing cloth. Every 10 days, a fixed amount of water was evenly sprayed, and the pile was turned over again to maintain the moisture content of the tea leaves at 20–25%. During the PF process, the temperature measured in the center and on both sides of the tea pile needed to be maintained at 50–55 °C. It is worth noting that the pile needs to be turned to avoid dehydration in case the temperature is too high. Finally, tea samples with PF durations of 0, 10, 20, 30, 40, 50, and 60 days were collected (abbreviated as Day 0, Day 10, Day 20, Day 30, Day 40, Day 50, and Day 60). Each group was set up with five biological replicates and stored at −80 °C for subsequent analyses.

### 2.3. Sensory Quality Evaluation of Tea Samples

Ten well-trained evaluation experts (4 males and 6 females, aged 25 to 55 years) assessed and scored the sensory quality following the Chinese Tea Sensory Evaluation Procedure GB/T 23776-2018 [18] in five aspects: appearance of tea leaves, color, aroma, taste of tea infusion, and leaf bottom, with scoring coefficients of 20:15:25:30:10, respectively. The taste aspect focused on bitter, astringent, sweet, sour, and fresh flavors and was scored on a ten-point scale. The brewing procedure involved brewing 3 g of tea leaves with 150 mL of freshly boiled distilled water for 2 min to obtain the first infusion for color evaluation. Subsequently, the tea was brewed for an additional 5 min with 150 mL of fresh boiled distilled water to obtain the second infusion for the evaluation of taste and aroma.

### 2.4. Color Variation Determination of Tea Infusions

The color of the tea infusion was measured using a spectrophotometer (CM-5, Konica Minolta, Tokyo, Japan) and the CIE L* a* b* color space 124 system, as described by Mao et al. [19]. L* value represents the brightness of the change from black (−L*) to white (+L*), while a* and b* values represent the intensity of the red–green (+ red, −green) and yellow–blue tones (+yellow, −blue), respectively. The brewing procedure for tea broth was the same as in 2.3. The color determination was repeated 3 times for each sample.

### 2.5. Metabolites Identification by Ultra-Performance Liquid Chromatography–Tandem Mass Spectrometry (UPLC-MS/MS)

The non-targeted metabolomics techniques were used to uncover the chemical profiles of tea samples with different PF durations, following the method of Qian et al. [20], and set up three biological replicates. Firstly, sample extraction was carried out in the following procedure: The vacuum freeze-dried tea samples were ground into powder, and 100 mg of powder was precisely weighed and dissolved in 0.6 mL of 70% methanol extract. Subsequently, the mixture was refrigerated at 4 °C overnight with six vortexing cycles. The next day, the extract was centrifuged (14,000 rpm, 10 min), and the resulting filtrate, passed through a nylon needle microporous filter membrane (SCAA-104, 0.22 μm pore size; ANPEL, Shanghai, China), was used for UPLC-MS/MS analysis.

The chromatograms were obtained using UPLC (Shim-pack UFLC SHIMADZU CBM30A, Shanghai, China) coupled with tandem mass spectrometry (MS/MS, Applied Biosystems 4500 QTRAP, Shanghai, China). The UPLC conditions were set as follows: The chromatographic column used was a Waters ACQUITY UPLC HSS T3 C18 (1.8 μm, 2.1 mm × 100 mm); The mobile phases A and B were ultrapure water and acetonitrile, respectively, both containing 0.04% acetic acid; The elution gradient started at 5% phase B, increased linearly to 95% over 10.00 min, held at 95% for 1 min, then decreased to 5% between 11.00 and 11.10 min, and remained at 5% until 14 min; The flow rate was set at 0.35 mL/minutes; The column temperature was maintained at 40 °C; The injection volume was 4 μL. The MS/MS conditions were as follows: electrospray ionization temperature set to 550 °C; mass spectrometry voltage at 5500 V; curtain gas at 30 psi; collision-activated dissociation parameter set to high; acquisition mode was the multi-reaction monitoring (MRM) mode in the data-dependent acquisition (DDA).

### 2.6. Statistical Analysis of Metabolites

The mass spectrometry data were processed using the Analyst 1.6.3 software, followed by comparison with MS/MS spectral information from Metware Database (MWDB), Human Metabolome Database (HMDB), and KEGG COMPOUND Database. Characteristic ions of each substance were identified through multiple reaction monitoring, and the signal intensity (CPS) of these characteristic ions was recorded by the detector. Integration and correction of the chromatographic peaks were performed using MultiaQuant software. Principal Component Analysis (PCA), hierarchical cluster analysis (HCA), orthogonal partial least squares-discriminant analysis (OPLS-DA), and calculation of Pearson’s Correlation Coefficient (r) were performed by R software 3.5.0 (http://www.r-project.org/ (accessed on 28 September 2023)). Prior to the PCA, the data were scaled by unit variance. The OPLS-DA model combining the fold change value (FC ≥ 2 or FC ≤ 0.5) and the variable importance in projection value (VIP ≥ 1) was applied to screen the differential metabolites. The VIP values were extracted from OPLS-DA results using the R package MetaboAnalystR. Graphs were plotted using GraphPad Prism 6.0 software (San Diego, CA, USA). A literature review was conducted to obtain taste characteristics and thresholds for differential metabolites.

### 2.7. Identification of Bacteria and Fungi DNA Sequences

The microbiomics techniques based on 16S rRNA and internal transcribed spacer (ITS) sequencing were used to understand the community structure of microorganisms and their dynamics in tea samples stored at −80 °C (as mentioned in 2.2), with five biological replicates set up for each group. Tea microbial DNA was first extracted and quality-controlled using the Trace Colony DNA Extraction Kit. The 16S rRNA sequences (The upstream primer was 341F with a sequence of 5′-CCTAYGGGRBGCASCAG-3′ and the downstream primer was 806R with a sequence of 5′-GGACTACNNGGGGTATCTAAT-3′) of bacteria and ITS sequences (The upstream primer sequence was 5′-CTTGTCATTTAGAGAAGTAA-3′ and the downstream primer sequence was 5′-GCTGCGTCTTCATCGATGC-3′) of fungi were then amplified, purified, and detected using the KAPA library amplification kit, Nextera XT Index Kit, KAPA Pure Beads Kit, and Qubit dsDNA HS kit. Finally, the samples were sent to Majorbio-Bio-Pharm Technology Co., Ltd. (Shanghai, China) for sequencing using the Illumina NovaSeq platform. Operational Taxonomic Units (OTUs) with 100% identity were clustered using the Uparse algorithm (Uparse v7.0.1001, http://www.drive5.com/uparse/ (accessed on 20 September 2023)), referring to the Silva database (http://www.arb-silva.de/ (accessed on 20 September 2023)) and Unite database (https://unite.ut.ee/ (accessed on 20 September 2023)) for species annotation.

### 2.8. Correlation Analysis of Metabolomics and Microbiomics

The correlation between the metabolomes and microbiomes was calculated using the cor function of R software, and the significance test was performed using the corPvalueStudent function of the weighted gene co-expression network analysis (WGCNA) package. Data with correlation |r| ≥ 0.8 and *p*-value < 0.05 were selected, and chord diagrams were plotted using the circlize package.

## 3. Results and Discussion

### 3.1. Quality Characteristics of Tea Samples in Different PF Duration

**Figure 1:**
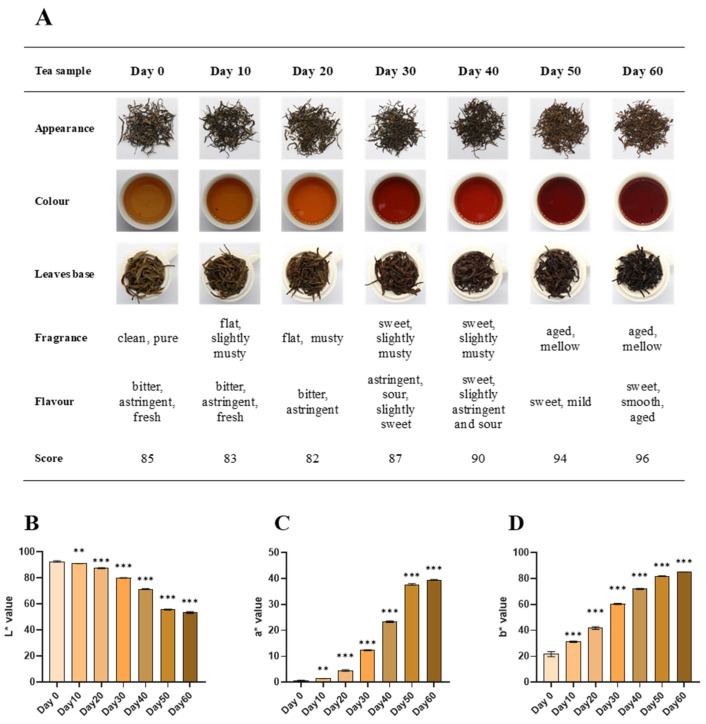
Quality characteristics of tea samples. (**A**) Sensory quality evaluation results, including five aspects of tea (appearance of tea leaves and leaf base, color, aroma, and taste of tea infusion) and overall scores. (**B**) L* values of tea infusions. (**C**) a* values of tea infusions. (**D**) b* values of tea infusions. Compared to Day 0, **: *p* < 0.01, ***: *p* < 0.001.

The sensory quality of tea samples in seven groups was assessed, and the results are shown in Figure 1A. Day 0 exhibited dark brown-green dry tea with tightly knotted strands; a yellow–green and bright infusion color; a clean and pure fragrance; a noticeable bitter, astringent, and fresh flavor; a yellow–green and uniform leaf base, with an overall quality score of 85. Day 10 and Day 20’s dry teas were not obviously different from Day 0. Day 10 had a dark yellow–green and still bright tea infusion; a flat and slightly musty aroma; a slightly reduced bitter, astringent, and fresh flavor; a dark yellow–green and uniform leaf base, scoring 83. Day 20 displayed an orange-yellow and bright infusion color, a distinctly musty scent, an astringent taste with a notable bitter aftertaste, and a dark yellow–green and uniform leaf base, scoring 82. Day 30 dry tea was dark brown with adhering microorganisms; the rope was still tightly knotted; the color of the tea infusion was orange-red and bright; the aroma was sweet and slightly musty; the taste was astringent with sourness and slight sweetness; the leaf base was brown-green and even, scoring 87. Day 40’s dry tea and leaf base were not visibly different from Day 30; the color of the tea infusion was dark red and bright; the aroma was sweet and slightly musty; the taste was sweet and slightly astringent and sour, scoring 90. Day 50 dry tea had a dark auburn color with tightly knotted strands, a red-rich and bright tea infusion, an aged and mellow aroma, a sweet and mild taste, and a dark red–brown leaf bottom, and the score was 94. Day 60’s dry tea and leaf base showed no noticeable improvement compared to Day 50, with a red–brown and bright tea infusion, a pure aged aroma, a sweet and smooth taste, with a distinctive aged flavor of dark tea, and the score was 96. Day 60 had the highest overall score and the best organoleptic quality among the seven groups of tea samples, and according to the changes in quality, especially in taste, the entire PF process could be divided into the early stage (Day 0–Day 20), the middle stage (Day 30–Day 40), and the late stage (Day 50–Day 60).

Based on the analysis of the chromaticity values, it was found that the tea broth of Day 0 had high brightness (L* = 92.66), which decreased significantly with the extension of PF duration, and the L* value of Day 60 was 53.42 (Figure 1B). Compared with Day 0 (a* = 0.62 and b* = 21.72), the a* and b* values of tea broth increased significantly with duration, with a* and b* values of 39.53 and 85.16 for Day 60, respectively (Figure 1C and 1D). The results showed that the red and yellow colors of tea were significantly enhanced after PF. This is consistent with the findings in the sensory evaluation, supporting the transformation of dark tea infusions from yellow–green to red–brown.

As for the different taste characteristics (Table 1), the astringency and freshness of the tea samples declined continuously throughout the PF process, compared with Day 0; the bitterness slightly increased in the early stage, then consistently decreased; the sourness rose rapidly in the early stage, decreased in the middle stage but remained at a high level, and continued to decrease in the late stage; while sweetness gradually increased with PF duration. Within the tea market, sweetness is usually preferred by consumers, while bitterness and astringency are often deemed undesirable [21]. Li et al., found that after a 40-day PF, the taste of Pu-erh tea changed from bitter and strong to sweet, mellow, stale, and pure [22]. Consistent with their research, with the prolongation of the PF duration, the astringency and bitterness of the single-cultivar large-leaf tea samples were significantly reduced, and the sweetness gradually increased, with the optimal effects observed at a PF duration of 60 days.

### 3.2. Metabolite Profiles During Dark Tea PF Process

#### 3.2.1. Dynamic Changes in Overall Metabolites

**Figure 2:**
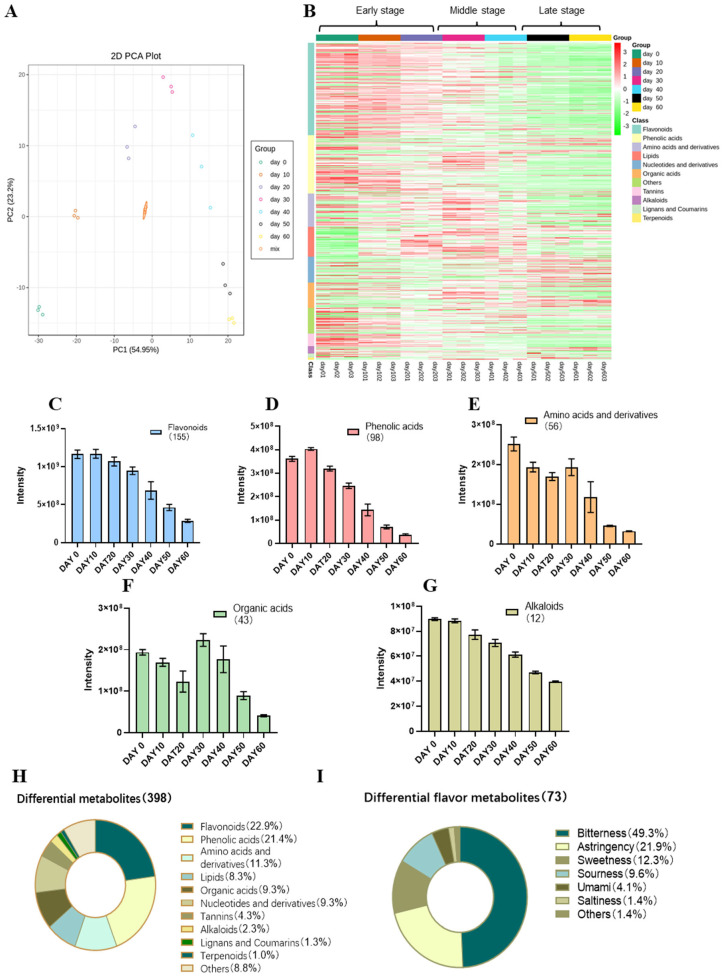
Profiles of tea metabolites. (**A**) PCA score plot of tea samples (sample MIX was the QC sample). (**B**) Clustering heat map of overall metabolites. (**C**) Peak area intensities of flavonoids. (**D**) Peak area intensities of phenolic acids. (**E**) Peak area intensities of amino acids. (**F**) Peak area intensities of organic acids. (**G**) Peak area intensities of alkaloids. (**H**) Classification chart of differential metabolites. (**I**) Classification chart of differential taste metabolites.

The quality control analysis (QC) was first performed. QC samples were prepared from a mixture of tea samples, which were used to analyze the reproducibility of the samples under the same treatments [21]. The obtained total ion current diagrams are shown in Figure S1, demonstrating a high overlap of the ion current curves and good signal stability, which is important for ensuring the reproducibility and reliability of the data. The PCA results are presented in Figure 2A, revealing a noticeable separation between different sample groups, indicating significant variation in metabolites with the prolongation of PF duration. The first two principal components (PC1 = 54.95%, PC2 = 23.2%) accounted for 78.15% of the total variance. Metabolites in the early, middle, and late stages could be clearly distinguished in PC1, suggesting distinguishable metabolite profiles among the three PF stages.

A total of 532 metabolites were annotated based on the metabolic database (Table S1). To understand the effect of PF duration on the type and content of tea metabolites, the metabolites were classified into 11 subsets, including flavonoids (155), phenolic acids (98), amino acids and derivatives (56), lipids (50), organic acids (43), nucleotides and derivatives (43), tannins (21), alkaloids (12), lignans and coumarins (7), terpenoids (4), and others (43). The clustering heat map (Figure 2B) revealed the changes in the contents of various metabolites during the PF process. Compared to Day 0, flavonoids and ellagitannins both consistently decreased with longer PF duration. Most phenolic acids reached their highest levels at Day 0, with some increasing abruptly at Day 20, but the majority continued to decrease in the last two PF stages. Amino acids and their derivatives, and organic acids followed an overall trend of first rising and then falling. Most amino acids, derivatives, and organic acids peaked on Day 30. Most alkaloids peaked on Day 0 and decreased as the PF duration progressed, while some showed opposite trends. The contents of nucleotides and their derivatives, and terpenoids almost continuously increased throughout the PF duration. The taste of tea infusion is usually associated with the levels of flavonoids, phenolic acids, amino acids, organic acids, and alkaloids [21]. Peak area intensities (Figure 2C–G) showed that the total levels of flavonoids, phenolic acids, amino acids, organic acids, and alkaloids on Day 60 were 24.7%, 10.3%, 12.7%, 21.0%, and 44.2% of Day 0, respectively. Therefore, among all 532 metabolites, flavonoids, phenolic acids, amino acids and their derivatives, and organic acids were more abundant and significantly decreased with the prolongation of the PF duration, which might contribute more to the taste changes in the PF process.

#### 3.2.2. Dynamic Changes in Differential Metabolites

To identify significantly differentially changed metabolites in the PF process, OPLS-DA plots were generated for tea samples from adjacent PF durations (Figure S2A–F). Validation of the model for each subgroup (Figure S2G–L) indicated that the R2Y and Q2 scores were both greater than 0.9, demonstrating the stability and reliability of the model. According to the OPLS-DA model (FC ≥ 2 or FC ≤ 0.5 and VIP ≥ 1), 398 differential metabolites were identified in total (Table S2). These metabolites were categorized into 11 subsets (Figure 2H), including flavonoids (91), phenolic acids (85), amino acids and derivatives (45), organic acids (37), nucleotides and derivatives (37), lipids (33), tannins (17), alkaloids (9), lignans and coumarins (5), terpenoids (4), and others (35).

**Figure 3:**
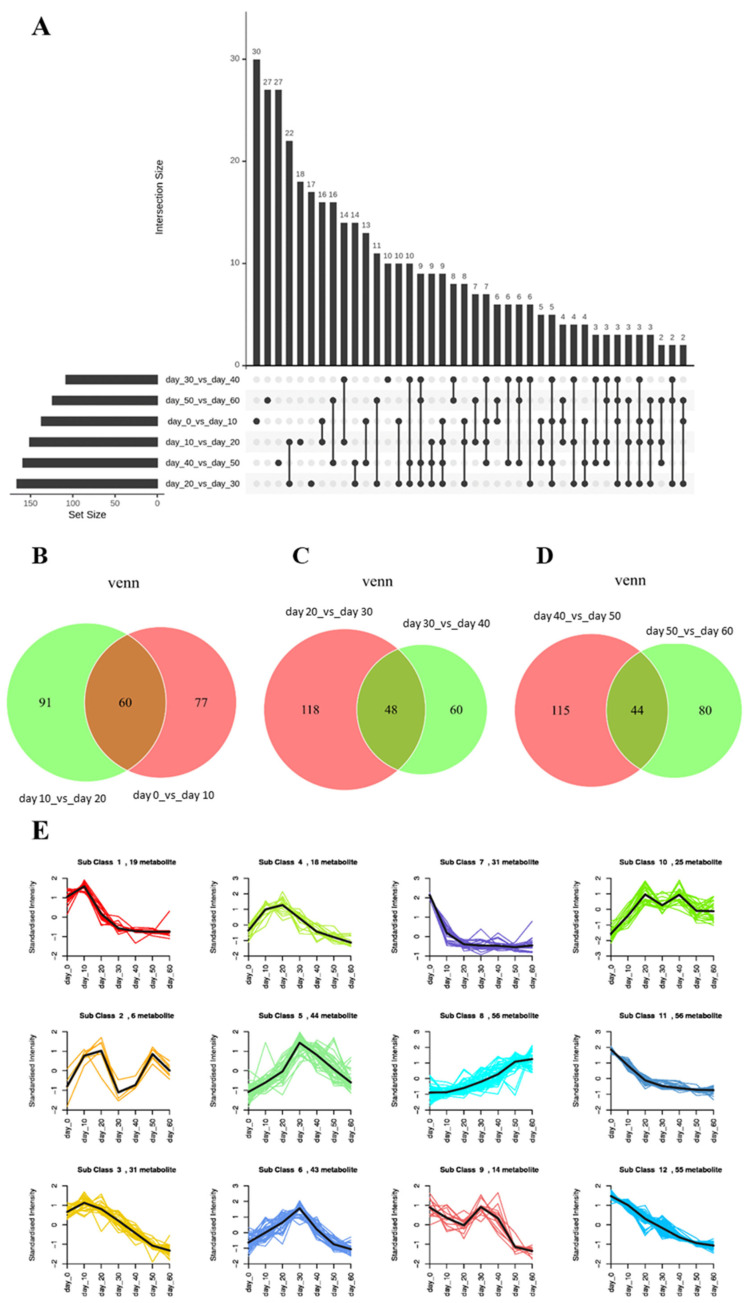
Profiles of differential metabolites. (**A**) Upset plot for overall layout of differential metabolites during PF process. (**B**) Venn diagram of differential metabolites during the early stage. (**C**) Venn diagram of differential metabolites during the middle stage. (**D**) Venn diagram of differential metabolites during the late stage. (**E**) K-means cluster plots of differential metabolites.

To understand the overall changes in the 398 differential metabolites, the layout of differential metabolites throughout the PF process and each stage was examined. The upset plot of shared differential metabolites obtained is shown in Figure 3A, and no shared differential metabolites were found in six subgroups, possibly due to the succession of microbial communities in tea during the PF process, resulting in changes in metabolites influenced by microbial communities as well. The Venn plots (Figure 3B–D) showed that there were 60 shared differential metabolites in the early stage, 48 shared differential metabolites in the middle stage, and 44 shared differential metabolites in the late stage, indicating the presence of certain shared differential metabolites in each stage, suggesting that these metabolites changed uniformly during this stage.

In order to further investigate the trends of each differential metabolite in the PF process, a k-means analysis was performed, and the results are shown in Figure 3E and Table S2. All differential metabolites were classified into 12 subclasses, and based on the changes in their content, they could be further categorized into four trends corresponding to the changes in taste characteristics: (i) 142 differential metabolites (Subclass 7, 11, and 12) tended to decrease continuously along the PF duration; (ii) the levels of 68 differential metabolites (Subclass 1, 3, and 4) elevated in the early stage and consistently declined in the last two stages; (iii) 126 differential metabolites (Subclass 5, 6, 9, and 10) mainly increased in the early stage, decreased in the middle stage but still at a high level, and continued to decrease in the late stage; (iv) 56 differential metabolites (Subclass 8) increased consistently as the PF duration progressed.

#### 3.2.3. Dynamics of Taste Differential Metabolites and Screening of Key Taste Metabolites

The taste of 398 differential metabolites and their oral thresholds were queried based on their CAS numbers, and the 73 differential metabolites that had oral flavor were summarized in Table 2. These taste metabolites belonged to 8 subsets, including amino acids and derivatives (18), nucleotides and derivatives (15), phenolic acids (10), organic acids (10), flavonoids (8), tannins (4), alkaloids (1), and others (7). They were classified according to their taste characteristics (Figure 2I), revealing that the differential components were mainly bitter substances (49.3%), followed by astringent (21.9%) and sweet (12.3%) substances. These components may contribute to the significant changes in bitterness, astringency, and sweetness of the tea samples during the PF process.

According to Table 2, among the astringent-dominant taste metabolites, theaflavin-3-gallate, theaflavin-3′-gallate, theaflavin 3,3′-digallate, L-tryptophan, procyanidin C1, rutin, and (-)-epicatechin gallate (ECG) belonged to trend i (Subclass 7, 11, and 12); while L-theanine, ferulic acid, methyl gallate (MG), protocatechuic acid (PCA), vanillic acid, pyrocatechol, caffeic acid, and GA were categorized under trend ii (Subclass 1, 3, and 4). Among the bitter-dominant metabolites, those belonging to trend i (Subclass 7, 11, and 12) were nicotinamide, 6-aminocaproic acid, L-valine, L-phenylalanine, and salicin; those belonging to trend ii (Subclass 1, 3, and 4) were adenosine, guanosine, epigallocatechin gallate, gallocatechin gallate, L-(-)-tyrosine, L-tyramine, chlorogenic acid, neochlorogenic acid, and L-epicatechin (EC); and those belonging to trend iii (Subclass 5, 6, 9, and 10) were histamine, cytosine, adenine, guanine, 7-methylxanthine, cytidine, Phe-Phe, N-glycyl-L-leucine, glycylisoleucine, glycyl-L-proline, L-isoleucine, α-Aminocaproic acid, L-(+)-lysine, and 3-hydroxybutyrate. The sweet-dominant metabolites salicylic acid, terephthalic acid, D-sorbitol, dulcitol, and α-hydroxyisobutyric acid were of trend iv (Subclass 8). The sour metabolites were anchoic acid, 4-hydroxybenzoic acid, L-(+)-tartaric acid, 2-methylsuccinic acid, and L-ascorbic acid, mostly belonging to trend iii (Subclass 5, 6, 9, and 10). The umami metabolites cyclic AMP, L-aspartic acid, succinic acid, and γ-aminobutyric acid with the mouth-drying sensation were all of trend i (Subclass 7, 11, and 12).

Of these 73 taste differential metabolites, 31 metabolites (Subclass 1, 3, 7, 8, 9, 11, and 12) showed significant changes in their contents on Day 60 compared to Day 0. So, they might be the key taste metabolites in the formation of the high-quality taste of dark tea. These 31 potential key taste metabolites included 26 astringent or bitter metabolites, such as L-valine, L-tryptophan, L-phenylalanine, 4-aminobenzoic acid, salicin, rutin, (-)-ECG, nicotinamide, theaflavin-3-gallate, theaflavin-3′-gallate, theaflavin 3,3′-digallate, procyanidin C1, 6-aminocaproic acid, L-(-)-tyrosine, L-isoleucine, L-theanine, α-aminocaproic acid, L-(+)-lysine, L-tyramine, adenosine, guanosine, epigallocatechin gallate, gallocatechin gallate, L-EC, chlorogenic acid, and neochlorogenic acid, all significantly decreased after the PF; and 5 sweet metabolites, including salicylic acid, terephthalic acid, D-sorbitol, dulcitol, and α-hydroxyisobutyric acid, increased after the PF.

#### 3.2.4. Contribution of Compounds and Key Taste Metabolites to Taste Formation in Single-Cultivar Large-Leaf Dark Tea

Exploring the changes in tea composition and taste metabolites during the PF process is crucial for understanding the foundation of taste development in dark tea. Bitter substances usually accompany astringent flavors, predominantly stemming from polyphenols in green tea, of which flavanols have the highest content, and catechins are the main representatives [23]. Throughout the conversion from green to dark tea, the most notable changes occur in catechins, with a dramatic decrease in their content. Flavonoids, another significant contributor to astringency and bitterness in dark tea, typically have very low taste thresholds and degrade during microbial fermentation [24]. Previous studies on black tea have shown a decrease in catechins and theaflavins (such as GCG, ECG, GA, EC, and TF-3-G) after PF, leading to a significant decrease in astringency [7]. Additionally, EGCG’s content was significantly correlated with the strength of green tea astringency, with a unique wrinkled astringent and bitter flavor compared to other catechins [21]. In this study, the total levels of flavonoids and ellagitannins both consistently decreased with longer PF duration, among which theaflavin-3-gallate (TF-3-G), theaflavin-3′-gallate (TF-3′-G), theaflavin 3,3′-digallate (TF-3,3′-DG), and ECG were key taste metabolites (trend i). Similarly, MG, PCA, GA, epigallocatechin gallate (EGCG), gallocatechin gallate (GCG), and EC gradually decreased in the last two stages (trend ii). These compounds contain various catechins and theaflavins with ester structures and have extremely low astringency and bitterness thresholds (Threshold ≤ 230 mg/kg), consistent with the previous findings. They primarily contribute to the reduction of bitterness and astringency in the tea samples.

In addition, phenolic acids also significantly contribute to tea taste. Previous studies [25] have suggested that the reduction of phenolic acids, such as chlorogenic acid and neochlorogenic acid, contributes to the transition from tea astringency to mellowness. Phenolic acid ethyl esters (protocatechuic, ferulic, and vanillic acid ethyl esters) activate bitter taste receptors (TAS2Rs) and contribute to bitter flavor [26]. In our study, most phenolic acids gradually decreased in the last two PF stages. Salicin, ferulic acid, vanillic acid, pyrocatechol, caffeic acid, chlorogenic acid, and neochlorogenic acid had very low thresholds of bitterness or astringency (Threshold ≤ 198 mg/kg), might likely play significant roles in bitterness and astringency changes. Furthermore, some bitter amino acids and nucleotides also showed a decline after the PF process, including L-tryptophan, L-valine, L-phenylalanine, L-(-)-tyrosine, L-tyramine, adenosine, and guanosine, although their thresholds were generally higher (Threshold: 800~4250 mg/kg), suggesting a lesser contribution to the bitter taste. In the study by Sun et al. [27], the removal of bitter amino acids did not result in differences in the taste intensity of tea soup, confirming their minimal impact on the bitter taste.

Differences in sugar and organic acid content significantly affect sweetness, with organic acids mitigating sourness when mixed with sugar [28]. Previous research has shown a positive correlation between sorbitol content and perceived sweetness intensity, surpassing other carbohydrates or total sugars [29]. In our study, the increased content of sweet metabolites might be the reason for the sweetness of tea soup. Salicylic acid, with a low threshold (Threshold: 414 mg/kg), and D-sorbitol, with a higher threshold (Threshold: 6160 mg/kg) and significant variation in content, may be representative compounds influencing sweetness.

Other taste characteristics besides bitterness, astringency, and sweetness also changed during the PF process, especially sourness, but their appearance indicated an insufficient PF duration [10]. Organic acids are a group of acids associated with sour taste. In this research, the organic acids increased abruptly at Day 30 and maintained a high level in the middle stage. This could be the reason for the higher sourness of the tea samples in the middle stage. Sour metabolites, such as anchoic acid, 4-hydroxybenzoic acid, L-(+)-tartaric acid, 2-methylsuccinic acid, and L-ascorbic acid had low thresholds (Threshold ≤ 276 mg/kg), making them the main contributors to the sourness of tea infusion, and most of which are organic acids. γ-Aminobutyric acid, which could cause dryness in the mouth, had an extremely low threshold (Threshold: 2.1 mg/kg) and decreased with PF duration. It was an important contributor to the mellowing and smoothness of tea samples. Changes in freshness might be related to the reduction of cyclic AMP, L-aspartic acid, and succinic acid. The only salty metabolite, L-glutamine, was a representative substance affecting the salty taste of dark tea.

In conclusion, 31 taste differential metabolites showed significant changes after the PF process, among which catechins and theaflavins with ester structures, EC, MG, PCA, GA, salicin, chlorogenic acid, and neochlorogenic acid showed a significant decrease after PF, which were key taste metabolites contributing to the reduction of astringency and bitterness in dark tea after the PF. Salicylic acid and D-sorbitol with sweetness increase after the PF, aiding in the formation of a sweet taste.

### 3.3. Comparison of Microbial Community Structures in Different PF Duration

**Figure 4:**
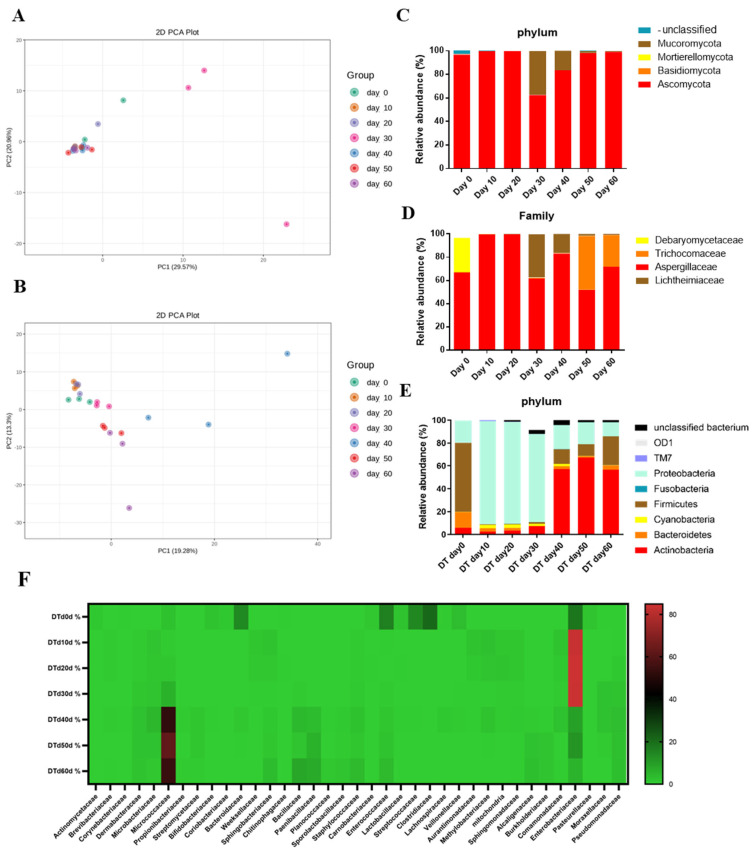
Comparison of microbial community structures. (**A**) PCA of fungi. (**B**) PCA of bacteria. (**C**) Relative abundance column cumulative plots of fungi at the phylum level. (**D**) Relative abundance column cumulative plots of fungi at the family level. (**E**) Relative abundance column cumulative plot of bacteria at the phylum level. (**F**) Relative abundance heat map of bacteria at the family level.

There were numerous microorganisms involved in the PF process, whose sequences are shown in Table S3. The PCA of fungi (Figure 4A) shows that the fungal community structure was relatively similar across different samples, except for Day 30; Day 0 and Day 60 were slightly different from the other samples, suggesting that the PF process had certain effects on the fungal community structure. The PCA of bacteria (Figure 4B) shows that the bacterial community could be distinguished between each group of samples, indicating significant changes in the bacterial community structure with the duration of PF. Furthermore, the early stage, middle stage, and late stage could be distinguished.

Fungi at the phylum level (Figure 4C) reveal that the fungal communities throughout the PF process were primarily composed of Ascomycota, Basidiomycota, Mortierellomycota, and Mucoromycota. Ascomycota was the predominant fungus throughout the PF process, with 96.72% on Day 0, peaking at 99.94% in the early stage, decreasing to 62.48% on Day 30, and then rapidly increasing in the last two stages to 99.01% on Day 60. Mucoromycota also showed a significant increase during the middle stage, reaching 37.31% abundance on Day 30, followed by a gradual decrease in the last two stages. At the family level (Figure 4D), the fungal communities were mainly comprised of Aspergillaceae, Trichocomaceae, Debaryomycetaceae, and Lichtheimiaceae. Both Aspergillaceae and Trichocomaceae, belonging to Ascomycota, showed distinct trends, with Aspergillaceae peaking at 99.72% in the early stage and declining in the last two stages, while Trichocomaceae showed a sharp increase in the late stage. Lichtheimiaceae, a part of Mucoromycota, followed a trend similar to Mucoromycota. The bar-cumulative plot of bacterial species at the phylum level (Figure 4E) indicates that the bacterial communities were primarily composed of Actinobacteria, Bacteroidetes, Cyanobacteria, Firmicutes, and Proteobacteria. Proteobacteria dominated the first 30 days of the PF process, peaking at 90.12% on Day 10 and gradually decreasing to 12.54% on Day 60. Actinobacteria became dominant in the last 30 days, with its abundance increasing with PF duration (2.56%~67.27%). Firmicutes had the highest abundance on Day 0 (60.22%) but grew slowly throughout the PF process, showing a trend similar to that of Actinobacteria (0.35%~25.26%). At the family level (Figure 4F), Enterobacteriaceae of Proteobacteria dominated the first 30 days, while Micrococcaceae of Actinobacteria dominated the last 30 days.

In summary, among the fungal communities in the PF process, Ascomycota was the dominant phylum, with Aspergillaceae and Trichocomaceae as the dominant families. Among the bacterial communities, Actinobacteria and Proteobacteria were the dominant phyla, with Micrococcaceae and Enterobacteriaceae as the dominant families. These microbial compositions likely have a significant influence on the changes in astringency, bitterness, and sweetness of dark tea.

### 3.4. Potential Contribution of Microorganisms to Dark Tea Taste

**Figure 5:**
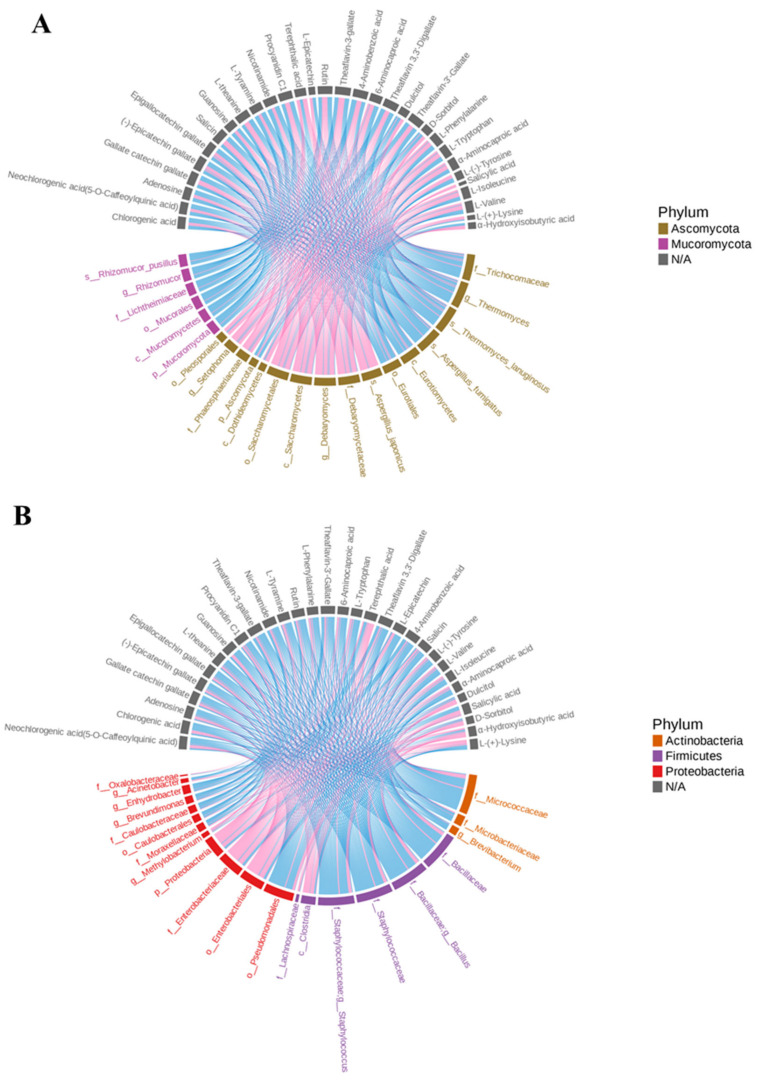
Correlation analysis of microbial communities and 31 significantly differential taste metabolites. (**A**) Correlation chord chart of fungi. (**B**) Correlation chord chart of bacteria. Pink color indicates positive correlation, blue color indicates negative correlation.

To better understand how the environmental microbiota affected the taste quality of dark tea through mycorrhizal metabolism and co-metabolism with tea in the PF process, the 31 significantly differential taste metabolites screened above were analyzed with differential microorganisms for Spearman-rank correlation analysis. Correlation chord plot (Figure 5A) and correlation coefficients (Table S4) for fungi revealed that Ascomycota was correlated with all 31 taste metabolites (*p*-value < 0.05), with higher correlations for dulcitol and D-sorbitol (correlation coefficients: 0.75 and 0.74), which were sweet in taste. On further exploring the correlation results of the fungi at the family level versus the genus level, these key metabolites were found to be correlated with *Aspergillus* of Aspergillaceae (*p*-value < 0.05) and were significantly correlated with *Thermomyces* of Trichocomaceae (*p*-value < 0.01). The correlation chord plot (Figure 5B) and correlation coefficients (Table S5) for bacteria showed that Micrococcaceae of Actinobacteria had a significant correlation (*p*-value < 0.01) with 31 metabolites, of which the correlation with the bitter and astringent neochlorogenic acid and chlorogenic acid were highest (both correlation coefficients of 0.94). To our surprise, the non-dominant phylum Firmicutes had even more significant correlations (*p*-value < 0.01). Further analyzing the correlations of families and genera under this phylum revealed that *Bacillus* of Bacillaceae and *Staphylococcus* of Staphylococcaceae exhibited conforming correlations with the phylum levels. *Bacillus* showed more significant correlations with TF-3-G, GCG, EGCG, TF-3′-G, and procyanidin C1 (correlation coefficients: 0.85~0.87), while *Staphylococcus* correlated with rutin and (-)-ECG (correlation coefficients: 0.86~0.84), which had bitter and astringent flavors.

During the PF process, tea components and taste metabolites underwent a series of different biotransformation reactions due to microorganisms, which in turn changed the taste of tea soup [30]. *Aspergillus* is a major genus in various dark teas and is involved in their component transformation [31]. It can produce protease, tannase, and various hydrolytic enzymes, which play an important role in changing the bitter and astringent taste of dark tea [32]. The results of our study are similar: *Aspergillus* could reduce the bitterness and astringency of tea samples by reducing the bitter and astringent metabolites. *Bacillus* is a promising microbial preparation for improving the quality of certain fermented foods [33]. It produces a variety of digestive enzymes, such as protease and amylase, which promote the breakdown of large molecules into smaller ones and can transform the taste components of sufu [34]. Previously, *Bacillus* was found to hydrolyze ester catechins, with a dramatic decrease in ester catechins and a significant increase in non-ester catechins in dark tea fermentation [35], which was consistent with the present study. *Staphylococcus* is generally regarded as a spoilage microorganism in food and is significantly correlated with reduced amino acid content in soy sauce [36]. In this study, it was found that it may be associated with a significant reduction in some bitter and astringent substances, especially ECG. Micrococcaceae are generally believed to be involved in the desirable reactions that occur during dry-fermented sausage maturation, such as peroxidative decomposition, protein hydrolysis, and lipolysis [37]. Our study found that it may significantly reduce phenolic acids, especially neochlorogenic acid and chlorogenic acid, which deserves further study.

In conclusion, *Aspergillus*, *Thermomyces*, *Bacillus*, *Staphylococcus*, and Micrococcaceae had higher correlations with the 31 significantly different taste metabolites. *Aspergillus*, *Bacillus*, *Staphylococcus*, and Micrococcaceae might be the core functional microorganisms to reduce the bitter and astringent key taste metabolites, while *Aspergillus*, *Thermomyces*, *Bacillus*, and *Staphylococcus* contributed to increase the sweet metabolites.

However, the effects of microorganisms in dark tea are not always beneficial, as they may also produce toxins. Toxins such as aflatoxins, ochratoxin A, fumonisins, etc., were detected in dark tea before, but they usually originated from uncontrolled or unsuitable fermentation/storage conditions [38]. In a previous study, none of the 100 dark tea samples contained aflatoxin B1 at levels higher than the limit of detection [39]. Interestingly, previous studies have found that the tea matrix and certain tea microorganisms can prevent the production of mycotoxins [40]. Therefore, the quality and safety of dark tea can be assured by strict monitoring of fermentation and storage conditions.

### 3.5. Potential Taste Formation Mechanism Involving Microbial Interactions During the Dark Tea PF Process

In the early stage, the abundance of *Aspergillus*, *Bacillus*, and *Staphylococcus* elevated, and they could produce a variety of extracellular enzymes for the hydrolysis and transformation of catechins with ester structures, which would release the relevant catechins and GA as products, and these products could form phenolic acids after a series of reactions. *Bacillus* also could activate upstream flavonoid pathway genes as well as repress downstream catechin branch genes [41]. The combined action of these microorganisms might result in the degradation or conversion of some flavonoids and catechins (TF-3-G, TF-3′-G, TF-3,3′-DG, ECG) in the early stage, which in turn led to a decrease in the bitterness and astringency of the tea samples at this stage. This might also be the reason some catechins (EGCG, GCG, EC), GA, and phenolic acids (MG, PCA, GA, salicin, chlorogenic acid, and neochlorogenic acid) are higher at this stage. In the last two stages, *Aspergillus* remained at a high level, while that of *Bacillus* and Micrococcaceae increased. *Aspergillus* is also a key microorganism in the production of methyltransferases, which can catalyze the conversion of GA and other phenolic acids to methoxyphenolic compounds, thus reducing the content of phenolic acids and GA. Therefore, the synergistic effect of these microorganisms may cause the simultaneous decrease of some flavonoids, catechins, and phenolic acids (TF-3-G, TF-3′-G, TF-3,3′-DG, ECG, EGCG, GCG, EC, MG, PCA, GA, salicin, chlorogenic acid, and neochlorogenic acid) in the last two stages, leading to a continued reduction in the bitterness and astringency of the tea samples.

Contributing substances to the higher sweetness after PF were salicylic acid, D-sorbitol, terephthalic acid, dulcitol, and α-hydroxyisobutyric acid, which were mainly some sugars and organic acids. Correlation analyses pointed to the possibility that *Aspergillus*, *Thermomyces*, *Bacillus*, and *Staphylococcus* caused their gradual increase. *Aspergillus* and *Thermomyces* belong to Ascomycota. *Aspergillus* possesses a rich reservoir of carbohydrate-degrading enzymes, of which glycoside hydrolases and glycosyltransferases are important enzymes, promoting the formation of tea polysaccharides and water-soluble sugar [31,42]. *Thermomyces* also can degrade insoluble polysaccharides to soluble polysaccharides and correlate with a wide range of biologically active monosaccharides [12]. *Bacillus* and *Staphylococcus* belong to Firmicutes, having a high-efficiency carbohydrate degradation system, which can degrade or ferment indigestible dietary fiber and produce a large number of metabolites, benefiting host immunity and intestinal micro-ecological balance [43,44]. Throughout the PF process, the abundance of Ascomycota remained high, and that of Firmicutes gradually increased, which may contribute to the improvement of the key sweetness metabolites, thus enhancing the sweetness of dark tea.

## 4. Conclusions

In this study, a single-cultivar large-leaf raw tea material was used to make dark tea, and the tea quality, metabolic characteristics, and microbial communities of the dark tea with different PF durations were investigated, with a detailed exploration of the relationship between them in detail. With the prolongation of the PF duration, the astringency and bitterness of the tea samples were significantly decreased, the sweetness increased, and the effect was better at a PF duration of 60 days. The study showed that microorganisms significantly influenced the metabolic components of dark tea and promoted the formation of dark tea taste quality through metabolic processes and enzyme secretion during PF. This study deepens the understanding of the taste formation mechanism of single-cultivar large-leaf dark tea and provides a reference for optimizing the dark tea process to improve the quality of dark tea. In future research, the focus will be on functional microorganisms in single-cultivar large-leaf dark tea. By controlling the parameters of temperature, humidity and duration during pile-fermentation or by inoculation, the growth of functional microorganisms can be promoted to improve the taste quality of dark tea.

## Figures and Tables

**Table 1:** Oral intensity of five taste factors of tea samples.

Tea Sample	Day 0	Day 10	Day 20	Day 30	Day 40	Day 50	Day 60
Bitterness	3	4	5	4	2	1	-
Astringency	8	7	5	2	1	-	-
Sweetness	-	-	-	2	4	5	6
Sourness	-	2	4	4	3	-	-
Freshness	2	2	1	-	-	-	-

“-” represents that there is no noticeable oral flavor.

**Table 2:** Scores of different taste characteristics.

NO.	Compounds	Formula	Group	Class	CAS	Taste	Threshold (mg/kg)
1	Cyclic AMP	C_10_H_12_N_5_O_6_P	7	Nucleotides and derivatives	60-92-4	Fresh	32,900
2	D-Glucose 6-phosphate	C_6_H_13_O_9_P	7	Others	56-73-5	Sweet	360
3	Theaflavin-3-gallate	C_36_H_28_O_16_	7	Tannins	30462-34-1	Crinkle type astringent	10.742
4	Theaflavin-3′-Gallate	C_36_H_28_O_16_	7	Tannins	28543-07-9	Crinkle type astringent	10.742
5	Theaflavin 3,3′-Digallate	C_43_H_32_O_20_	7	Tannins	30462-35-2	Crinkle type astringent	11.284
6	L-AsparticAcid	C_4_H_7_NO_4_	11	Amino acids and derivatives	56-84-8	Fresh	80
7	L-Tryptophan	C_11_H_12_N_2_O_2_	11	Amino acids and derivatives	73-22-3	Bitter	1225
						Astringent	102
8	D-Serine	C_3_H_7_NO_3_	11	Amino acids and derivatives	312-84-5	Sweet	3150–4200
9	Nicotinamide	C_6_H_6_N_2_O	11	Others	98-92-0	Bitter	730–980
10	D-(+)-Sucrose	C_12_H_22_O_11_	11	Others	57-50-1	Sweet	7200
11	Procyanidin C1	C_45_H_38_O_18_	11	Tannins	37064-30-5	Bitter	347
						Crinkle type astringent	260
12	6-Aminocaproic acid	C_6_H_13_NO_2_	11	Organic acids	60-32-2	Bitter	3670–4200
13	L-Valine	C_5_H_11_NO_2_	12	Amino acids and derivatives	72-18-4	Bitter	1950
14	L-Phenylalanine	C_9_H_11_NO_2_	12	Amino acids and derivatives	63-91-2	Bitter	910
15	Salicin	C_13_H_18_O_7_	12	Phenolic acids	138-52-3	Bitter	83
16	Rutin	C_27_H_30_O_16_	12	Flavonoids	153-18-4	Astringent	1220–1830
17	(-)-Epicatechin gallate	C_22_H_18_O_10_	12	Flavonoids	1257-08-5	Bitter	200
						Crinkle type astringent	115
18	Succinic acid	C_4_H_6_O_4_	12	Organic acids	110-15-6	Sour	94
						Fresh	83
19	γ-Aminobutyric acid	C_4_H_9_NO_2_	12	Amino acids and derivatives	56-12-2	Dry mouth feeling	2.1
20	Adenosine	C_10_H_13_N_5_O_4_	1	Nucleotides and derivatives	58-61-7	Bitter	800–1600
21	Guanosine	C_10_H_13_N_5_O_5_	1	Nucleotides and derivatives	118-00-3	Bitter	4250
22	Epigallocatechin gallate	C_22_H_18_O_11_	1	Flavonoids	989-51-5	Crinkle type astringent	87
						Bitter	87–174
23	Gallocatechin gallate	C_22_H_18_O_11_	1	Flavonoids	4233-96-9	Crinkle type astringent	180
						Bitter	180
24	L-(-)-Tyrosine	C_9_H_11_NO_3_	3	Amino acids and derivatives	60-18-4	Bitter	725–1090
25	L-theanine	C_7_H_14_N_2_O_3_	3	Amino acids and derivatives	3081-61-6	Silky type astringent	1050
26	L-Tyramine	C_8_H_11_NO	3	Amino acids and derivatives	51-67-2	Bitter	274–343
27	Chlorogenic acid	C_16_H_18_O_9_	3	Phenolic acids	327-97-9	Sour	361
						Bitter	50
28	Neochlorogenic acid	C_16_H_18_O_9_	3	Phenolic acids	906-33-2	Bitter	50
						Sour	1000
29	L-Epicatechin	C_15_H_14_O_6_	3	Flavonoids	490-46-0	Bitter	230
						Crinkle type astringent	230
30	Anchoic Acid	C_9_H_16_O_4_	3	Organic acids	123-99-9	Sour	188
31	Ferulic acid	C_10_H_10_O_4_	4	Phenolic acids	1135-24-6	Crinkle type astringent	13
32	Methyl gallate	C_8_H_8_O_5_	4	Flavonoids	99-24-1	Astringent	42.688
33	Histamine	C_5_H_9_N_3_	5	Amino acids and derivatives	51-45-6	Bitter	1110–2220
34	4-Hydroxybenzoic acid	C_7_H_6_O_3_	5	Phenolic acids	99-96-7	Sour	276
						Bitter	1100–1660
						Astringent	92
35	Cytosine	C_4_H_5_N_3_O	5	Nucleotides and derivatives	71-30-7	Bitter	780–1000
36	Adenine	C_5_H_5_N_5_	5	Nucleotides and derivatives	73-24-5	Bitter	270–540
37	Guanine	C_5_H_5_N_5_O	5	Nucleotides and derivatives	73-40-5	Bitter	>760
38	7-Methylxanthine	C_6_H_6_N_4_O_2_	5	Nucleotides and derivatives	552-62-5	Bitter	100–200
39	Cytidine	C_9_H_13_N_3_O_5_	5	Nucleotides and derivatives	65-46-3	Bitter	3650–4860
40	Protocatechuic acid	C_7_H_6_O_4_	5	Flavonoids	99-50-3	Crinkle type astringent	31–32
41	L-(+)-Tartaric acid	C_4_H_6_O_6_	5	Organic acids	87-69-4	Sour	41
42	2-Methylsuccinic acid	C_5_H_8_O_4_	5	Organic acids	498-21-5	Sour	99
43	Phe-Phe	C_18_H_20_N_2_O_3_	6	Amino acids and derivatives	2577-40-4	Bitter	200–300
44	N-Glycyl-L-leucine	C_8_H_16_N_2_O_3_	6	Amino acids and derivatives	869-19-2	Bitter	4700
45	Glycylisoleucine	C_8_H_16_N_2_O_3_	6	Amino acids and derivatives	19461-38-2	Bitter	410
46	Glycyl-L-proline	C_7_H_12_N_2_O_3_	6	Amino acids and derivatives	704-15-4	Bitter	1030
47	Vanillic acid	C_8_H_8_O_4_	6	Phenolic acids	121-34-6	Crinkle type astringent	53
48	Pyrocatechol	C_6_H_6_O_2_	6	Phenolic acids	120-80-9	Bitter	198
						Astringent	99
49	Caffeic acid	C_9_H_8_O_4_	6	Phenolic acids	331-39-5	Crinkle type astringent	13
50	Gallic acid	C_7_H_6_O_5_	6	Flavonoids	149-91-7	Crinkle type astringent	46.2–50
						Bitter	>140
51	L-Ascorbic acid	C_6_H_8_O_6_	6	Others	50-81-7	Sour	120
52	3-Hydroxybutyrate	C_4_H_8_O_3_	6	Organic acids	300-85-6	Bitter	>10,400
53	L-Isoleucine	C_6_H_13_NO_2_	9	Amino acids and derivatives	73-32-5	Bitter	1310–1575
54	α-Aminocaproic acid	C_6_H_13_NO_2_	9	Amino acids and derivatives	327-57-1	Bitter	2360–2890
55	L-(+)-Lysine	C_6_H_14_N_2_O_2_	9	Amino acids and derivatives	56-87-1	Bitter	11,700–13,160
56	L-Glutamine	C_5_H_10_N_2_O_3_	9	Amino acids and derivatives	56-85-9	Salty	7300
57	2-Aminoisobutyric acid	C_4_H_9_NO_2_	9	Amino acids and derivatives	62-57-7	Sweet	515–1030
58	Salicylic acid	C_7_H_6_O_3_	8	Phenolic acids	69-72-7	Sweet	414
59	Terephthalic acid	C_8_H_6_O_4_	8	Phenolic acids	100-21-0	Sweet	>6640
60	Thymine	C_5_H_6_N_2_O_2_	8	Nucleotides and derivatives	65-71-4	Bitter	440–630
61	1,7-Dimethylxanthine	C_7_H_8_N_4_O_2_	8	Nucleotides and derivatives	611-59-6	Bitter	90–160
62	Hypoxanthine	C_5_H_4_N_4_O	8	Nucleotides and derivatives	68-94-0	Bitter	5990
63	1-Methylxanthine	C_6_H_6_N_4_O_2_	8	Nucleotides and derivatives	6136-37-4	Bitter	230–300
64	Uracil	C_4_H_4_N_2_O_2_	8	Nucleotides and derivatives	66-22-8	Bitter	<2800
65	Thymidine	C_10_H_14_N_2_O_5_	8	Nucleotides and derivatives	50-89-5	Bitter	480–730
66	D-Sorbitol	C_6_H_14_O_6_	8	Others	50-70-4	Sweet	6160
67	Nicotinic acid	C_6_H_5_NO_2_	8	Others	59-67-6	Bitter	2460–3800
68	Dulcitol	C_6_H_14_O_6_	8	Others	608-66-2	Sweet	8000
69	Theophylline	C_7_H_8_N_4_O_2_	8	Alkaloids	58-55-9	Bitter	110–160
70	2-Furanoic acid	C_5_H_4_O_3_	8	Organic acids	88-14-2	Astringent	18
71	Adipic Acid	C_6_H_10_O_4_	8	Organic acids	124-04-9	Sour	168
72	α-Hydroxyisobutyric acid	C_4_H_8_O_3_	8	Organic acids	594-61-6	Sweet	>10,400
73	Cytidylic acid	C_9_H_14_N_3_O_8_P	2	Nucleotides and derivatives	63-37-6	Fresh	2260

## Data Availability

The original contributions presented in the study are included in the article/Supplementary Material, further inquiries can be directed to the corresponding authors.

## Supplementary Materials

The following supporting information can be downloaded at: https://www.mdpi.com/article/10.3390/foods14040670/s1, Figure S1: Samples quality control analysis; Figure S2: Subgroups orthogonal partial least squares-discriminant analysis (OPLS-DA); Table S1: Overall metabolites; Table S2: Differential metabolites; Table S3: DNA sequences statistics; Table S4: Correlation statistics for fungi; Table S5: Correlation statistics for bacteria.
