# Supplementary material for: Effects of Pile-Fermentation Duration on the Taste Quality of Single-Cultivar Large-Leaf Dark Tea: Insights from Metabolomics and Microbiomics
Source: Foods. 2025 Feb 16;14(4):670. doi: 10.3390/foods14040670 (PMC11854364; doi:10.3390/foods14040670)
Supplement: Supplementary file 1 [file foods-14-00670-s001.zip › foods-3397736-Figures.pptx]

## Slide 1
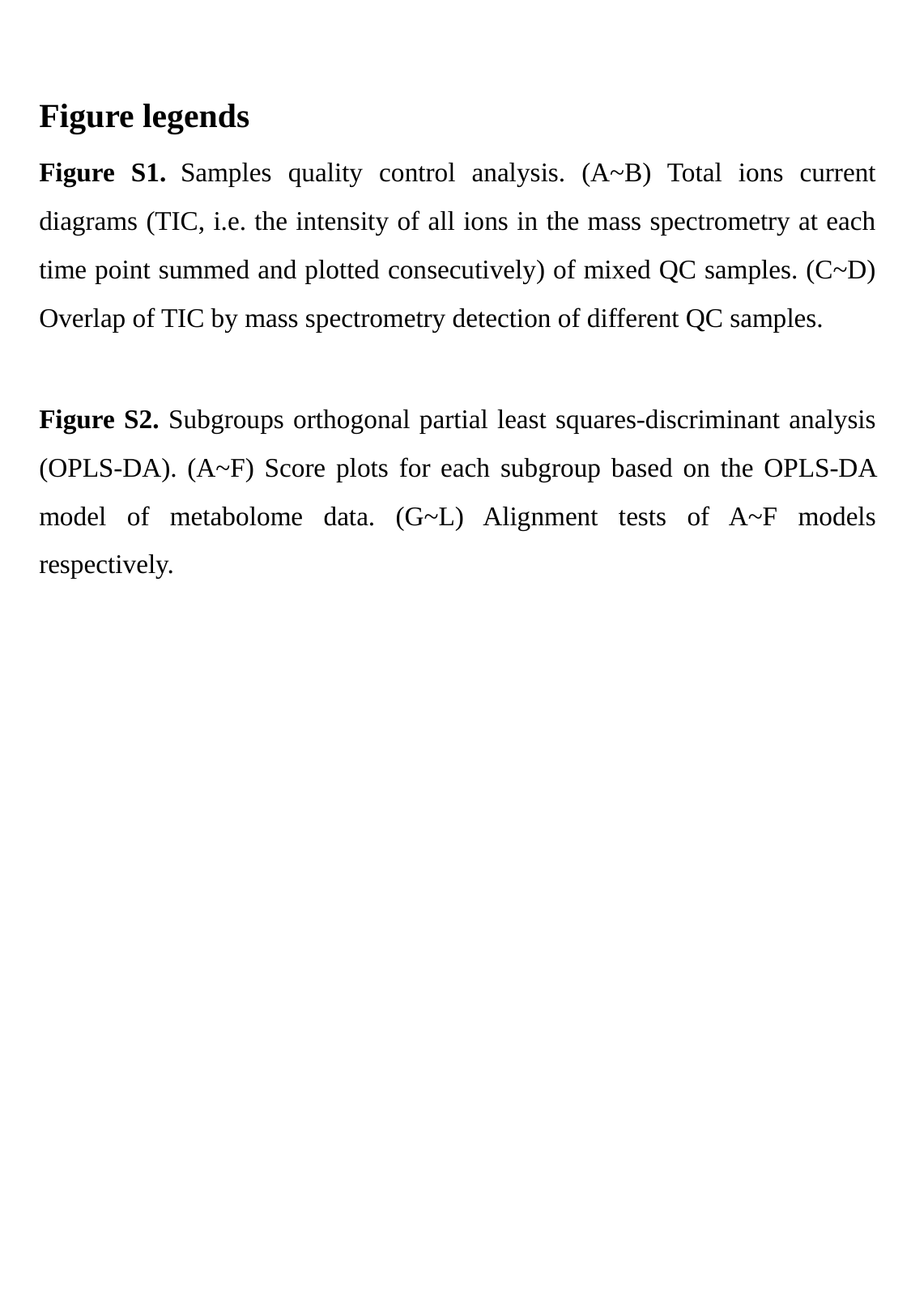

Figure legends
Figure S1. Samples quality control analysis. (A~B) Total ions current diagrams (TIC, i.e. the intensity of all ions in the mass spectrometry at each time point summed and plotted consecutively) of mixed QC samples. (C~D) Overlap of TIC by mass spectrometry detection of different QC samples.
Figure S2. Subgroups orthogonal partial least squares-discriminant analysis (OPLS-DA). (A~F) Score plots for each subgroup based on the OPLS-DA model of metabolome data. (G~L) Alignment tests of A~F models respectively.

## Slide 2
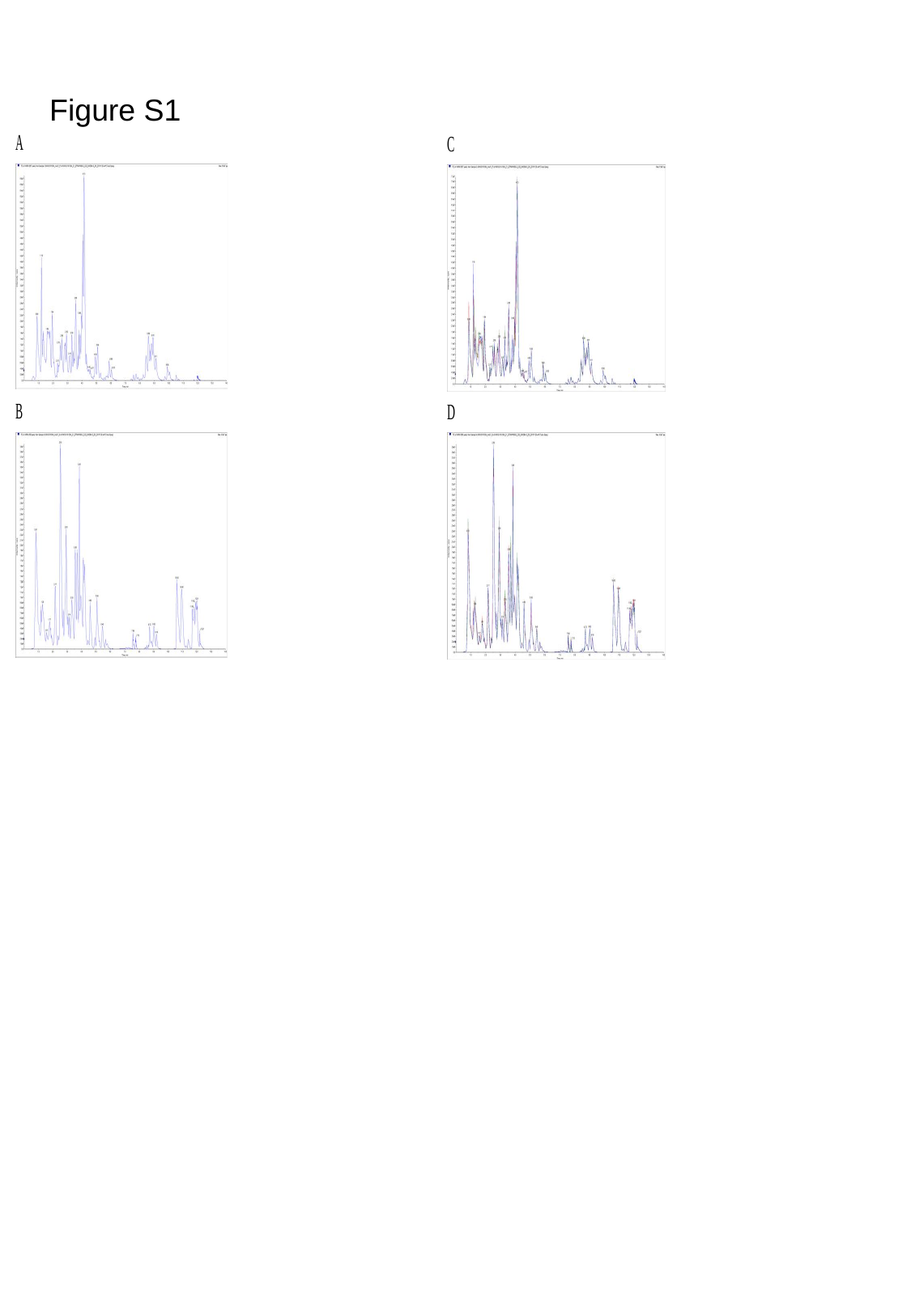

Figure S1

## Slide 3
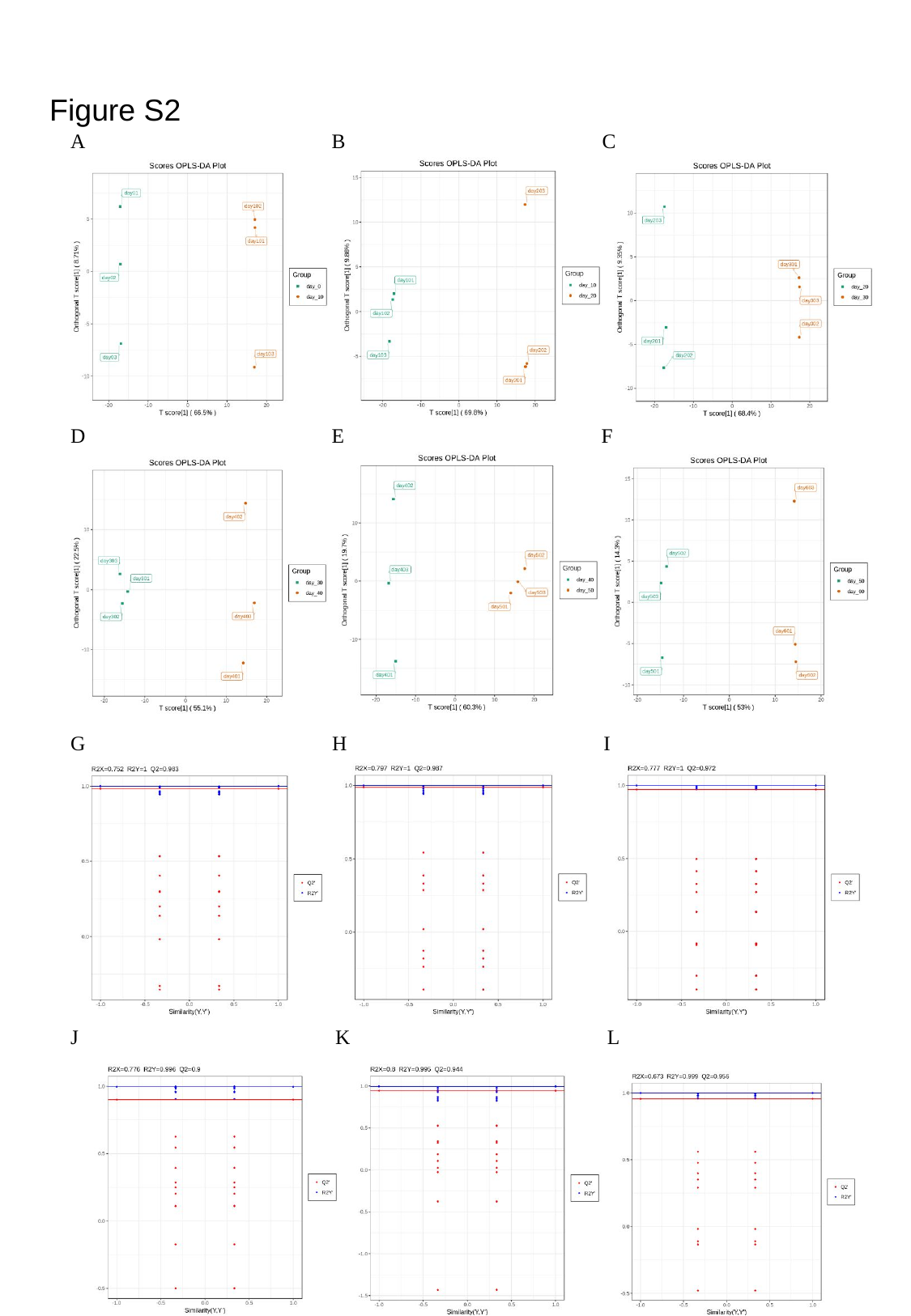

Figure S2
